# Conventional angiography findings in hemodynamically unstable patients with acute abdominal hemorrhage and a negative CT bleeding study

**DOI:** 10.1186/s42155-020-00112-7

**Published:** 2020-04-20

**Authors:** Amy C. O’Brien, Gerard M. Healy, Nicholas Rutledge, Aishan Patil, Jeffrey W. J. McCann, Colin P. Cantwell

**Affiliations:** 1 Department of Radiology, St Vincent’s University Hospital, Dublin, Ireland; 2 Department of Medicine, St Vincent’s University Hospital, Dublin, Ireland; 3 University of Glasgow Medical School, Glasgow, UK; 4 UCD School of Medicine & Medical Science, Radiology Department and University College Dublin, St. Vincent’s University Hospital, Elm Park, Dublin 4, D04 T6F4 Ireland

**Keywords:** Humans; Hemorrhage; Hemodynamics; Tomography; X-ray computed; Angiography

## Abstract

**Background:**

CT bleeding study (CTA) is regularly requested in acute abdominal haemorrhage (AAH) with haemodynamic instability by clinical teams and interventional radiologists because CTA can; detect arterial bleeding at low rates of hemorrhage, accurately localize the bleeding point and characterize the etiology.

How best to manage an unstable patient who has an AAH with a haematoma and no acute vascular findings on CTA represents a difficult clinical scenario for treating physicians and Interventional Radiologists.

**Purpose:**

To review the conventional angiography (CA) findings and clinical outcome of hemodynamically unstable patients with AAH who had a preceding negative CTA.

**Materials and methods:**

All patients who were hemodynamically unstable and underwent CTA and CA for acute arterial abdominal hemorrhage at our institution between 01/01/2010 and 31/12/2017 were identified. Patients with obstetric, penetrating trauma, abdominal aortic or venous sources of hemorrhage were excluded. Patients who had a negative CTA before CA were included. Patient medical records were reviewed for clinical outcome.

**Results:**

In the study period 160 hemodynamically unstable patients underwent 178 CA procedures. 155 CA procedures were preceded by CTA. 141 CTAs demonstrated active bleeding or an abnormal artery. 14 CTAs in 13 patients demonstrated hematoma but no acute bleeding (mean age = 56-years; M:F, 12:1). Eight of the 14 CA studies demonstrated: active bleeding (*n* = 4), pseudoaneurysm (*n* = 1) or a truncated artery (*n* = 3). Cases of renal hemorrhage demonstrated a significantly higher proportion of false negative CTA studies (36%). Selective (*n* = 8) or empiric (*n* = 4) embolization was performed in twelve cases. All patients stopped bleeding and there were no mortalities.

**Conclusion:**

In a cohort of hemodynamically unstable patients, 57% (8/14) of cases with no acute vascular findings on CTA demonstrated a source of hemorrhage on CA. The false negative rate of CTA was significantly higher for renal tract hemorrhage compared to other sites of bleeding.

## Background

Acute abdominal hemorrhage (AAH) can be intraluminal (upper or lower gastrointestinal bleeding) or extra luminal (bleeding into the parenchyma of an organ, peritoneal cavity or abdominal wall). Acute upper and lower gastrointestinal hemorrhage (GI) treatment guidelines typically suggest endoscopy is the first line investigation (Strate and Gralnek 2016), with CT angiography (CTA), nuclear medicine red cell labelled imaging and conventional angiography (CA) appropriate as second line investigations (ACR appropriateness guidelines). The recently published British Society of Gastroenterology guidelines have advised CTA as first line investigation in hemodynamically unstable patients, prior to endoscopy, CA or surgery (Oakland et al. 2019). Prior studies of CTA in acute GI hemorrhage suggest that patients with a negative CTA are likely to stop bleeding without intervention (Chan et al. 2015; Foley et al. 2010).

There are no specific guidelines for investigating extra luminal hemorrhage in the abdomen outside of the setting of blunt and penetrating trauma (the American Trauma Life Support (ATLS) guidelines). CTA is regularly requested in AAH with haemodynamic instability by clinical teams and interventional radiologists because CTA can; detect arterial bleeding at low rates of hemorrhage, accurately localize the bleeding point, characterize the etiology (E.G. bleeding ulcer), display the vascular anatomy, determine the arterial access site and aid selection of angiographic equipment.

How best to manage an unstable patient who has an AAH with a haematoma and no acute vascular findings on CTA represents a difficult clinical scenario for treating physicians and Interventional Radiologists.

The purpose of our study is to review the CA findings and clinical outcome of hemodynamically unstable patients with acute abdominal hemorrhage who had a preceding negative CTA.

## Materials and methods

**Fig. 1:**
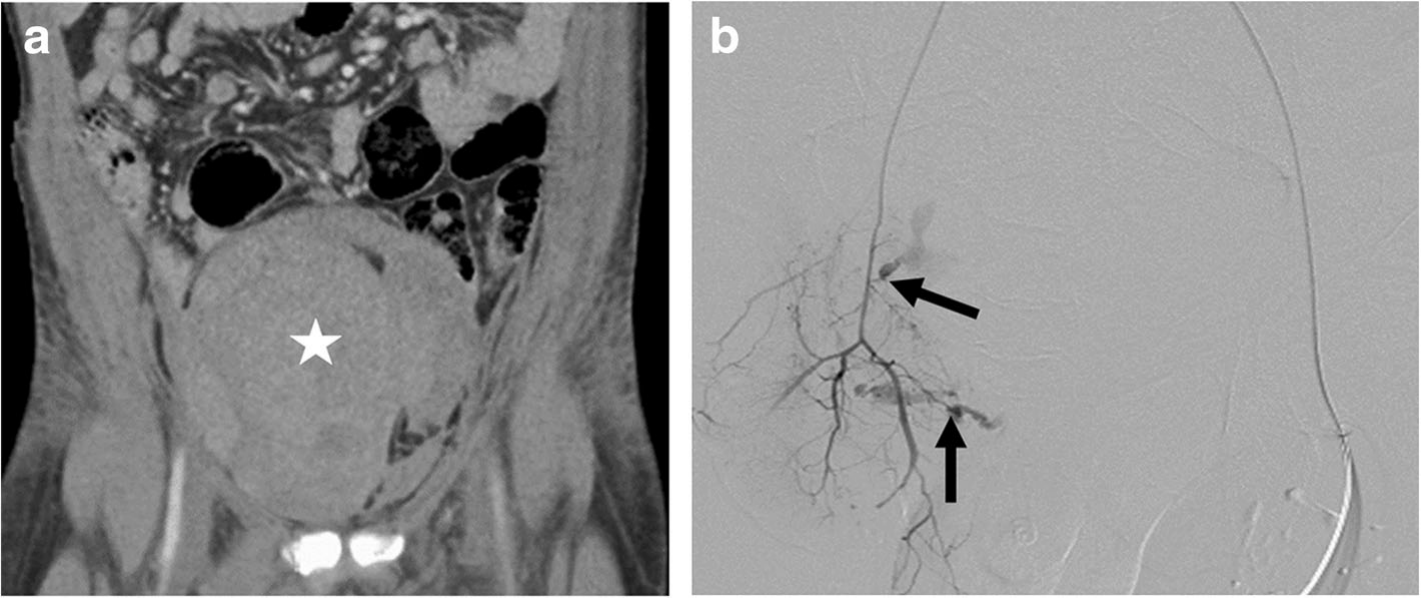
**a**. Coronal reconstruction of a negative CTA. A large extraperitoneal hematoma (star) is seen in the pelvis. No active extravasation of contrast or abnormal vessel was seen on CTA. **b**. Posterior-anterior projection digital subtraction angiogram of the obturator branch of the anterior division of the right internal iliac artery. This was performed using an end-hole catheter inserted from a left common femoral artery access sheath. Active hemorrhage is seen from two branch arteries (black arrows)

This retrospective study was approved by our institutional research board. The requirement for patient consent was waived. During the treatment period all GI bleeding was investigated primarily by endoscopy. All acute extraluminal hemorrhage was investigated primarily with CTA. Hemodynamically stable patients with imaging features of recent bleeding (hematoma present) but no acute vascular findings on CTA would undergo inpatient clinical surveillance. Patients who remain hemodynamically unstable after negative CTA and resuscitation would undergo CA despite a normal CTA (Fig. 1).

### Patients search

All CA procedures for acute abdominal hemorrhage performed from 01/01/2010 to 31/12/2017 were identified using a search of the Radiology Information System (RIS). A database entry was generated recording; demographics, suspected aetiology, endoscopy performed, if CTA was performed, time of CTA completion, presence of acute vascular findings on CTA, reasons for not performing CTA, hemodynamic stability at the time of CTA (hemodynamic instability was defined as a sustained systolic blood pressure less than 90 mmHg and heart rate greater than 100 beats/min (Loggers et al. 2017)), hematoma location at CTA (upper and lower GI hemorrhage, parenchymal, peritoneal and abdominal wall bleeding), time of commencing CA, findings at CA, embolic material used and clinical success. Obstetric, penetrating trauma, abdominal aortic and venous bleeding cases were excluded. Only hemodynamically unstable patients who had a negative CTA for acute vascular findings were included in the study.

### CT technique

All CTA studies were performed using a 64-slice CT scanner (Somatom Cardiac CT, Siemens Healthineers, Erlangen, Germany). No oral contrast was administered. Following acquisition of a non-contrast CT abdomen and pelvis range extended from the diaphragm to the symphysis pubis. 150 ml of low-osmolar iodinated contrast (340 mg/ml Iodine or higher concentration) was administered by power injector intravenously at 4 ml/sec. Arterial-phase imaging was triggered using bolus tracking in the abdominal aorta at a density threshold of 125 Hounsfield units. The portal-venous-phase was acquired 25 s later and/or the delayed phase at three minutes after the arterial phase. All CT scans were reconstructed and archived with contiguous thin sections of 1 mm thickness and routine acquisition and archiving of coronal and sagittal reconstructions. CT scans were primarily interpreted by an abdominal imaging fellowship trained attending.

Active bleeding was defined as the detection of high-density contrast accumulation in the bowel lumen or an abdominal hematoma between the non-contrast and contrast-enhanced CTA phases. Acute vascular findings included the presence of active bleeding, pseudoaneurysm, truncated/irregular artery or arterio-venous fistula. A hematoma alone on CTA was defined as a negative CTA study.

### Angiographic technique

Angiography was performed using one of two single panel c-arm interventional angiography systems (Artis Q or Axiom DTA angiography suite, Siemens Healthineers, Erlangen, Germany). All procedures were performed via 5-Fr introducer sheath inserted using ultrasound and fluoroscopic guidance into the right common femoral artery. Selective and non-selective digital subtraction angiography was performed with hand or power injection via the suspected bleeding artery (e.g. renal artery) or celiac, superior mesenteric and inferior mesenteric arteries in GI bleeding using 5-Fr selective angiographic catheters. In some cases, coaxial 1.7–2.3-Fr microcatheters were used for sub-selective angiography. If a hematoma was present on CTA in a vascular territory then non-selective and selective angiography of the vascular territory was performed. If a patient had a percutaneous drain in position and this was the suspected site of bleeding then the drain would be removed over a guidewire and angiography repeated.

Embolization was performed using metal coils or gelatin sponge as a slurry or torpedo selected at the discretion of the operator depending on the vascular anatomy and the lesion site. If the CA was negative, empirical embolization of an arterial territory could be performed at the discretion of the operator.

### Imaging review

All negative CTA studies and subsequent CA procedures were reviewed retrospectively by a fellowship-trained abdominal imaging, vascular IR attending and IR fellow in consensus.

A positive CA was defined as demonstration of active arterial bleeding, presence of a pseudoaneurysm, arterio-venous fistulas or irregular/truncated arteries.

### Clinical follow-up

Patient clinical notes were reviewed for clinical outcome, transfusion need, hemodynamic stability and complications after the CA. Clinical success was defined as absence of symptoms or signs of ongoing bleeding and no further transfusion need.

### Patients identified

**Fig. 2:**
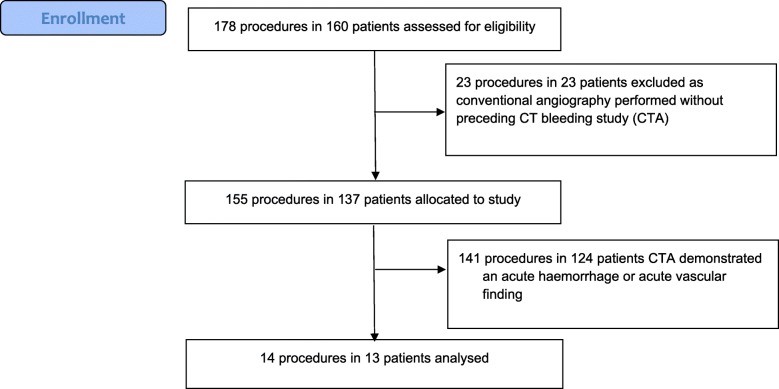
Flow diagram of patients with hemodynamic instability who underwent conventional angiography (CA) procedures for acute abdominal arterial hemorrhage during the study

160 patients with hemodynamic instability underwent 178 CA procedures for acute abdominal arterial hemorrhage during the study (Fig. 2). 23 patients were referred directly to the angiography suite for CA without a CTA, since there was a known source of arterial bleeding, recurrent bleeding from a known artery or significant hemodynamic instability that in the view of the operator would not allow a delay for CTA. 155 CAs were preceded by CTA. All CTAs were reviewed and confirmed that 140 CTAs demonstrated an acute vascular finding. One CTA was categorized as a positive study as a pseudoaneurysm was identified on retrospective imaging review. 14 CTAs in 13 patients were confirmed on review as negative for acute vascular findings. CA was performed within 2.5 h of CTA in all cases. The demographics and site of bleeding for these 14 cases are listed in Table 1.

**Table 1:** Review of the 14 negative CTA studies

Patient Number	Age (years)	Sex	Clinical presentation	Conventional Angiogram finding	Embolization treatment delivered
1	51	Male	Upper gastrointestinal bleed	Normal	Empiric treatment with Gelfoam
2	29	Male	Upper gastrointestinal bleed	Active bleeding	Gelfoam and metal coils
3	68	Male	Upper gastrointestinal bleed	Normal	Empiric treatment with Gelfoam
4	71	Male	Upper gastrointestinal bleed	Normal	Empiric treatment with metal coils
5	56	Male	Upper gastrointestinal bleed	Abnormal arteries	Gelfoam and metal coils
6	43	Male	Upper gastrointestinal bleed	Active bleeding	Metal coils
7	75	Male	Lower gastrointestinal bleed	Normal	Nil
8	83	Male	Lower gastrointestinal bleed	Normal	Nil
9	34	Male	Extraperitoneal pelvic haematoma post blunt trauma	Active bleeding	Metal coils
10	37	Male	Haematuria post renal biopsy	Pseudoaneurysm	Metal Coils
11	72	Male	Renal cell carcinoma and haematuria	Active bleeding	Metal Coils
12	48	Female	Haematuria post nephrostomy insertion	Active bleeding	Metal Coils
13 –Episode 1	39	Male	Haematuria after percutaneous nephrolithotomy	Normal	Empiric treatment with metal coils
13 – Episode 2	39	Male	Haematuria after percutaneous nephrolithotomy	Abnormal arteries	Metal coils

All eight of the acute GI hemorrhage cases underwent endoscopy prior to CTA and CA. For the six upper GI bleeding cases; clips were applied to a bleeding point in one case, a bleeding points was identified but could not be adequately controlled in two cases and no bleeding point was found in three patients (but clot was visualised within the upper GI lumen). Three patients with upper GI bleeding were found to have treatable acute vascular findings at CA. For the two lower GI bleeding cases, one demonstrated hematoma within the colon but no bleeding point. In the other case, a bleeding point was identified at the hepatic flexure and metallic clips were applied.

### Data analysis

Data was analysed using SPSS version 23. (IBM, New York, USA). Using CA as the gold standard to define a case a positive or negative for an acute vascular finding, contingency tables were created comparing the CTA and CA results for the six anatomical categories of: upper GI, lower GI, renal, liver, abdominal wall and ‘other extra-luminal site’ bleeding (Table 2). Then 2 × 6 categorical tables were created for each of the following categories: false negative, true negative, false positive and true positive. Fishers exact test was then applied to these categorical tables, to compare the proportion of false negative etc. cases between the six anatomical categories. The Fisher’s exact test was chosen because of the expected high proportion of values < 5 within the Tables. A *P* value of < 0.05 was considered significant.

**Table 2:** CTA studies categorised by true/false positive/negative and bleeding site

	Upper gastro-intestinal bleeding (*n* = 34)	Lower gastro-intestinal bleeding (*n* = 26)	Renal (*n* = 11)	Liver (n = 17)	Abdominal / pelvic wall (*n* = 17)	Other extra-luminal sites (*n* = 50)	p
False negative	3 (9%)	0	4 (36%)	0	1 (6%)	0	< 0.001
True negative	3 (9%)	2 (8%)	1 (9%)	0	0	0	0.09
False positive	4 (12%)	6 (23%)	0	2 (12%)	1 (6%)	9 (18%)	0.46
True positive	24 (71%)	18 (69%)	6 (54%)	15 (88%)	15 (88%)	41 (82%)	0.18
Sensitivity	89%	100%	60%	100%	94%	100%	
Specificity	43%	25%	100%	NA	NA	NA	
Positive predictive value	85%	75%	100%	88%	94%	82%	
Negative predictive value	51%	100%	20%	NA	NA	NA	

*NA* Not assessed

## Results

### Imaging review

The CA was positive for an acute vascular finding in eight of the 14 negative CTA cases. In those eight cases the following findings were demonstrated: active bleeding (5 patients), pseudoaneurysm (1) and abnormal artery (2).

### Clinical follow-up

Arterial embolization was performed in twelve of the fourteen CA procedures. In all eight cases with a target acute vascular lesion on CA, this was treated with embolization. Six cases had no acute vascular lesion on CA. In four of these negative CA cases empiric embolization was performed. Two remaining negative CA resulted in no embolization (Table 1).

One patient underwent two procedures. This patient had renal tract hemorrhage post nephrolithotomy. He had a negative CTA, but remained hemodynamically unstable and therefore underwent initial negative CA and empiric embolization using two 6 mm Nester coils deployed in the accessory left renal artery. He represented with frank haematuria on day 12 post embolization. A CTA showed a small hematoma in the left renal pelvis and bladder but no active bleeding. He then had a second CA which demonstrated irregularity of a lower pole renal artery branch vessel, which was treated with embolization using 3 mm vortex micro coils.

None of the patients required further blood transfusion in the follow up period. All patients clinically improved and demonstrated no signs of further haemodynamic instability or bleeding. Therefore, the clinical success rate of positive CA with embolization was 100% (8/8). The clinical success rate of negative CA with empiric embolization was 75% (3/4). No complications or deaths occurred within 30 days of CA.

### Data analysis

The proportion of true/false negative and positive cases is detailed in Table 2. Regarding the false negative cases, there was a significant difference between the groups (*p* < 0.001), with 4/11 (36%) of CTA studies false negative for renal heamorrhage, compared to 3/34 (9%) for upper GI bleeding and 1/17 (6%) for abdominal / pelvic wall bleeding cases. There were no false negative CTA studies in patient with liver hemorrhage (*n* = 17) or for the other sites of extra-luminal hemorrhage (peripancreatic, splenic, mesenteric). There was no significant difference between groups when comparing the proportions of true negative, false positive or true positive cases (Table 2).

## Discussion

In this cohort of hemodynamically unstable patients with acute arterial abdominal hemorrhage, 57% (8/14 patients) of those with no acute vascular findings on CTA demonstrated a treatable target lesion on CA. Therefore, absence of acute vascular findings on CTA should not exclude consideration for CA and embolization if the patient is unstable.

In cases of luminal GI hemorrhage, our results demonstrate a higher number of false negative CTAs for upper GI hemorrhage compared to lower GI hemorrhage. This is in line with previously published literature, which has shown that upper GI hemorrhage has a significantly higher false negative CTA rate and that in patients with a lower GI bleed (Chan et al. 2015).

Regarding extraluminal hemorrhage, cases of renal hemorrhage demonstrated the highest proportion of false negative CTA studies (36%) in our cohort. Three out of four of these patients were iatrogenic injuries due to a renal biopsy or nephrolithotomy. An article comparing CTA and CA for renal hemorrhage found that of fourteen patients who had negative results on CTA, nine (64%) were found to have treatable acute vascular lesions on CA, which were successfully treated with coil embolization (Mao et al. 2015). In the single case where we performed AE on two occasions, the injured artery in the renal parenchyma may have been in spasm and intermittently bleeding, hence not visualized on the initial CTA or CA. An empiric lower pole embolization was performed at the first AE procedure on an accessory renal artery.

There are limitations in our study; our small study population comprised a unique cohort of patients with AAH in a single centre, all of whom underwent CA and CTA. However, we did not capture all presentations to our centre with a clinical diagnosis of AAH, since some patients with GI hemorrhage will go straight to endoscopy for management at our centre, without a CTA. Therefore, it was not possible to calculate sensitivity of CTA for source of GI hemorrhage with our collected data. AAH is intermittent due to the body’s response to bleeding (including vessel spasm and vessel thrombosis). Therefore, comparison of two diagnostic tests is difficult when they are performed at two different time points.

## Conclusion

In a cohort of hemodynamically unstable patients, 57% (8/14) of cases with no acute vascular findings on CTA demonstrated a source of hemorrhage on CA. The false negative rate of CTA was significantly higher for renal tract hemorrhage compared to other sites of bleeding.

## Data Availability

All data generated or analyzed during this study are included in this published article.
